# Ultrafast Dynamics of Colloidal Copper Nanorods: Intraband versus Interband Excitation

**DOI:** 10.1002/smsc.202100103

**Published:** 2021-12-15

**Authors:** Benjamin T. Diroll, Soojin Jeong, Xingchen Ye

**Affiliations:** ^1^ Center for Nanoscale Materials Argonne National Laboratory 9700 S. Cass Avenue Lemont IL 60439 USA; ^2^ Department of Chemistry Indiana University 800 E. Kirkwood Avenue Bloomington IN 47405 USA

**Keywords:** coherent acoustic phonons, copper, electron−phonons, nanorods

## Abstract

Colloidal copper nanorods (NRs) display transverse and longitudinal localized surface plasmon resonances. The longitudinal localized surface plasmon modes are tunable through the near‐infrared electromagnetic radiation energies with NR aspect ratios. Visible and near‐infrared transient optical response of the copper NRs is investigated under excitation conditions spanning intraband and interband excitation (0.79−3.50 eV). In both the visible and near‐infrared regions, the spectral response of the samples under intraband excitation (<2 eV) differs substantially from their response under interband excitation (>2 eV). However, the timescale of the electron−phonon coupling estimated from pump fluence‐dependent measurements (*τ*_ep_) is less sensitive to excitation conditions than reports for gold. *τ*_ep_ shortens slightly from ≈616 fs with intraband excitation (at visible probe energies) to ≈565 fs with interband excitation. The observed dynamics correspond to an average sample electron−phonon coupling parameter varying across all conditions from 4.4 × 10^16^ to 6.4 × 10^16^ J m^−3^ K^−1^, which is similar to bulk copper. Furthermore, coherent acoustic phonons are observed for the longitudinal localized surface plasmon resonance with a range of oscillatory periods reflecting sample size dispersion.

## Graphical abstract

Colloidal copper nanorods show distinctive branching of the transient spectral response when photoexcited at interband versus intraband transitions, but little change in electron−phonon coupling times.

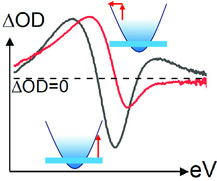

## 1 Introduction

First unambiguously identified by Faraday,^[^ ^1^ ^]^ metallic nanostructures are well known to support localized surface plasmon resonances (LSPRs), resulting from the collective oscillation of electrons on the nanostructure surface. The high extinction and environmental sensitivity of these LSPR features are integral to a diverse range of applications, including sensing^[^ ^2^, ^3^ ^]^ and diagnostics,^[^ ^4^ ^]^ medical therapies,^[^ ^5^ ^]^ optical circuitry^[^ ^6^, ^7^ ^]^ and switching,^[^ ^8^ ^]^ and chemical reactions.^[^ ^9^, ^10^ ^]^ LSPRs also offer a handle to examine the properties of the underlying material, such as thorough pump‐probe spectroscopy.^[^ ^11^ ^]^ The preponderance of existing work on nanostructures supporting LSPRs is focused on noble metal nanostructures and nanoparticles, with gold being the dominant metal in literature. For a variety of reasons including cost, spectral range, optical losses, temperature stability, and compatibility with end uses, there have been many efforts to expand the chemistry of plasmonic nanoparticles and nanostructures to other metals and doped semiconductors.^[^ ^12^, ^13^, ^14^ ^]^ As the lightest coinage metal, copper is a commodity metal which has similar bulk optical properties to gold and therefore represents a promising material for many of the same applications, with advantages particularly in compatibility with microelectronics fabrication. At the same time, the synthetic development of copper nanoparticles, particularly shape control, considerably lags that of gold and silver.

This work presents results on the dynamic optical properties of colloidal copper nanorods (NRs), which have a longitudinal LSPR feature, which is tunable through the infrared spectral window by synthetically adjusting the NR aspect ratio.^[^ ^15^ ^]^ A large class of the application space for plasmonic materials leverages not only their static optical properties, but also the dynamical behavior of excited electrons in time through light illumination. Ultrafast optical characterizations are critical for benchmarking certain fundamental materials properties and applications, which require the transduction of light to heat, chemical reactions, or optical switching effects. Previous works have studied the dynamical optical properties of bulk copper,^[^ ^16^, ^17^, ^18^, ^19^, ^20^, ^21^, ^22^, ^23^, ^24^ ^]^ with a particular interest in ablation and melting and copper nanoparticles.^[^ ^24^, ^25^, ^26^, ^27^, ^28^ ^]^ Here, ultrafast pump‐probe spectroscopy is employed at different pump energies—spanning interband transitions at high energy to intraband transitions at low energy—to investigate the photothermal dynamics of copper NRs. Intraband and interband excitations are found to induce large differences in the spectral response of the copper NRs, both at visible wavelengths associated with the interband transitions and in the near‐infrared LSPR features. Intraband excitation generates a thermal response, in which the photoexcitation of free electrons results in a redshift of interband transitions similar to that which is observed in static heating experiments.^[^ ^29^ ^]^ In contrast, interband excitation induces a large bleaching of the interband transitions of the copper NRs. These results underline a distinct contrast with gold and silver, which show similar spectral responses under interband and intraband excitation^[^ ^30^ ^]^ and may explain distinctive photon energy‐dependent photochemistry of copper nanostructures.^[^ ^31^, ^32^ ^]^ At the same time, the different excitation schemes have relatively small effects on the observed dynamics and, implicitly, electron−phonon coupling. Across all sample pump and probe conditions, average electron−phonon coupling times varied from 450 to 730 fs, with corresponding average sample electron−phonon coupling parameters from 4.4 × 10^16^ to 6.4 × 10^16^ J m^−3^ K^−1^, similar to reported values in bulk copper. This is distinct from previous work on gold nanoparticles, in which large differences in the electron−phonon coupling dynamics are observed for the intraband versus interband excitation regimes.^[^ ^33^ ^]^ The near‐infrared response of the samples, resonant with their LSPR features, is complicated by photoselection effects of the pump excitation, inhomogeneity of aspect ratios, and the presence of oscillatory signals derived from coherent acoustic phonons.

## 2 Results and Discussion

### 2.1 Static Optical Properties of Copper NRs

**Figure 1:**
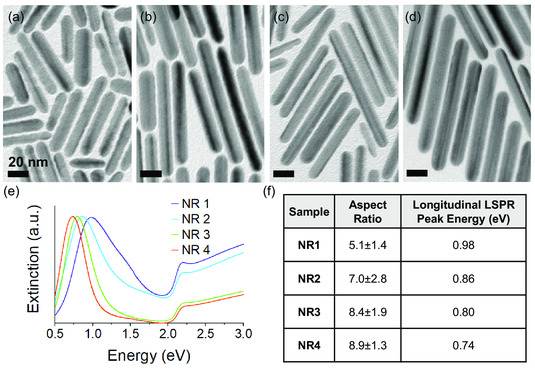
a−d) TEM images and e) extinction spectra of colloidal copper NR samples: a) NR1, b) NR2, c) NR3, and d) NR4 studied in this work. Note that this sample labeling convention is used throughout this article. f) Summary of average aspect ratios and longitudinal LSPR peak energies for different NR samples.

Copper NRs were synthesized by seed‐mediated synthesis, which yields pentatwinned NRs, such as those shown in the transmission electron microscopy (TEM) images in **Figure** **1**a–d.^[^ ^15^ ^]^ At the size scale of the samples used in this work (10 nm or greater), the electronic structure of the copper NRs is bulk like. To reduce the formation of copper oxides, which are prevalent in other nanoscopic copper systems,^[^ ^34^ ^]^ the samples were synthesized, purified, and measured under air‐free conditions. Similar to other anisotropic metal nanostructures, the extinction spectra of the copper NRs display two LSPR modes: a transverse LSPR similar to the LSPR observed in spherical copper nanoparticles^[^ ^25^, ^26^, ^28^ ^]^ near 2.1 eV and a longitudinal mode which depends on the aspect ratio of the NR (Figure 1e).^[^ ^15^ ^]^ In these samples, the aspect ratio was varied from 8.9 to 5.1, which generates a longitudinal LSPR feature of the NR ensembles ranging from 0.74 to 0.98 eV at the peak in the near‐infrared spectral region, covering wavelengths associated with telecommunications and part of the second near‐infrared transparency window of tissue. The transverse LSPR energy of copper NRs is insensitive to the size and size dispersion of the ensemble, whereas the longitudinal mode is highly sensitive to sample inhomogeneity. In addition to the plasmonic spectral feature, the samples also show the interband transitions of copper from the 3*d* to the 4*s* band, also starting near 2.1 eV, derived from the bulk band structure.^[^ ^29^, ^35^, ^36^, ^37^, ^38^ ^]^ Because the density of states of the *d*‐band is very high compared with energies near the Fermi level,^[^ ^39^ ^]^ the static and dynamic optical response of coinage metals at energies above the interband transition threshold is dominated by interband transitions.^[^ ^30^, ^35^, ^40^, ^41^ ^]^

### 2.2 Visible Transient Spectral Response

Previous works comparing optical dynamics and photothermal responses of metals and metal nanostructures have, in some cases, found divergent spectral and dynamical behavior between interband and intraband excitation (e.g., resonant with LSPR features).^[^ ^33^, ^42^, ^43^, ^44^ ^]^ The NR samples in this work represent excellent materials to examine this behavior for copper, as the large extinction of the longitudinal LSPR feature provides a strong optical absorption feature for excitations (as opposed to high reflectivity in the bulk films) and an excellent probe of transmittance changes. The copper NRs were examined by pump‐probe transient absorption spectroscopy using variable energy excitations as well as probe energies spanning visible and near‐infrared energies covering interband and LSPR intraband transitions (see Figure S1, Supporting Information). The pump intensity was maintained at modest levels (≤540 μJ cm^−2^) to avoid irreversible sample changes such as melting or ablation, which would have been apparent from the change in sample transmittance caused by agglomeration or sedimentation.

**Figure 2:**
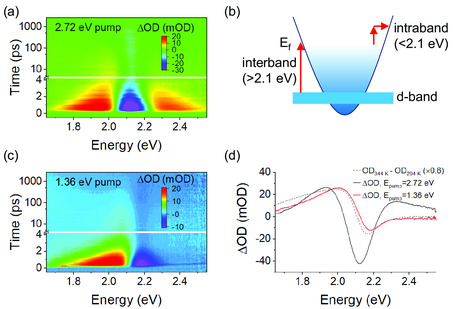
a,c) Map of the near‐infrared transient optical response of a copper NR sample under excitation with a) 2.72 eV pump photon energy and c) 1.36 eV pump photon energy. b) Cartoon of the band structure of copper showing intraband and interband transitions. d) Comparison of spectral line‐cuts at 500 fs pump‐probe delay for interband excitation (black) and intraband excitation (red) with static thermal difference absorption data shown in the gray dashed line. All data are taken from the same sample (NR3).

For the copper NRs studied here, the spectral response under pump‐probe experiments is strongly dependent upon the photon energy of the pump—specifically whether it photoexcites the free electrons of the LSPR modes (intraband) or the bound electrons of the interband absorptions. Two representative examples of interband and intraband photoexcitation at the same fluence (538 μJ cm^−2^) are shown in the transient extinction maps shown in **Figure** **2**a,b, respectively. Under interband excitation, shown with the cartoon in Figure 2c, promotion of *d*‐band electrons to the unoccupied states above the Fermi level (*E*_f_) results in a strong bleaching feature at 2.12 eV associated with the interband absorptions.^[^ ^19^, ^35^, ^36^, ^37^, ^38^ ^]^ Under interband excitation, at energies both higher and lower than the bleaching feature, the copper NRs showed induced absorption features, similar to reported transient spectra of silver^[^ ^30^ ^]^ and gold films or nanostructures.^[^ ^33^, ^45^, ^46^, ^47^ ^]^ Under intraband excitation at 1.36 eV pump photon energy shown in Figure 2c, the spectral response is quite different: the samples show a relatively strong photoinduced absorption feature at probe energies lower than 2.1 eV and a weak bleaching feature at high energies. To emphasize the robustness of the findings in this work, as well as the fact that the response is fundamental to the copper NRs as a class, rather than the specific NR samples, each sample of copper NRs is reported with the same spectral response, as represented in Figure 2. (See Figure S2, Supporting Information) Each of the samples showed a transition between the two responses at 1.76 eV, which shows a response similar to all other intraband excitation measurements, and 2.24 eV pump energy, which showed a response similar to all other interband excitation measurements. Small differences (30 meV, Figure S2, Supporting Information) in the energy of peak extinction changes were observed in the samples related to a similar magnitude of differences in the static extinction spectra. Earlier literature on the pump‐probe response of copper nanoparticles also suggests a transition from an intraband‐specific response to interband‐specific response near 2 eV. Under 2 eV photoexcitation, ultrafast spectral responses of copper nanoparticles resemble both of the spectral responses observed here under interband^[^ ^24^ ^]^ or intraband excitation.^[^ ^25^ ^]^ The pump fluence dependence of this response was also examined and shown in Figure S3, Supporting Information. Higher fluence slightly expands the energy range of transient signals, consistent with a larger perturbation of the electrons smearing about the Fermi level, as observed in static thermal difference and transient spectroscopic measurements on many metals. However, fluence does not substantively alter the differences observed under intraband and interband excitation conditions.

The response of the copper NRs under interband and intraband excitation is distinct from, for example, silver nanoparticles for which identical responses are reported for exciting in the near‐infrared (with weak extinction) and on resonance with the LSPR feature.^[^ ^30^ ^]^ The origin of these distinct responses is examined in Figure 2d by comparison of the transient extinction spectra (ΔOD) at a pump‐probe delay of 500 fs, shown with solid lines, with thermal differential absorption, which is shown with a dashed line. Previous examinations of the temperature‐dependent properties of copper show a similar thermal difference spectrum for bulk copper, arising chiefly due to a small redshift, broadening, and weakening of interband absorptions at elevated temperatures, as well as an increase in optical losses in the intraband region.^[^ ^19^, ^29^, ^48^ ^]^ In Figure 2d, it may be noted that the static differential extinction (here ΔOD = OD_344K_ − OD_294K_) showing the equilibrium thermal response is very similar to the transient optical response resulting from heating of electrons under intraband photoexcitation. On this basis, it may be asserted that intraband excitation (<2 eV pump photon energy) results in a thermal response, at least for pump‐probe delays of 500 fs or longer, in which electrons transiently reach an elevated effective temperature with properties which closely resemble those of a statically heated sample.

In contrast, the spectral response of the copper NRs under interband photoexcitation at pump‐probe delay of 500 fs differs considerably from the thermal response due to the electronic bleaching feature of the interband transitions. The two regimes of the spectral response suggest large differences in the distribution of electrons in the density of states of under interband and intraband photoexcitation regimes. In particular, the bleach feature at 2.12 eV observed under interband excitation is stronger and occurs at lower energy than the weaker bleach found under intraband excitation or in static thermal difference spectra in Figure 2d. The divergence of the interband pump‐probe response from the thermal difference spectrum (which reflects a small perturbation of electrons about the Fermi level) is an indication that under interband excitation, electrons and particularly holes in the copper NRs occupy states far from the Fermi energy. These energetic electrons and holes therefore may prove more reactive than in gold or silver, although holes in the *d*‐band are relatively localized and have low velocity.^[^ ^41^, ^49^ ^]^ Other literature is supportive of this conclusion. For example, earlier experiments on copper/metal oxide diodes have shown that interband excitation results in larger incident photon‐to‐current efficiency, attributed to the enhanced probability for photoexcited electrons to overcome the Schottky barrier at the metal−semiconductor interface.^[^ ^31^ ^]^ Using Raman techniques, it was also found that hot (nonequilibrium) electrons in copper are more reactive and abundant than in gold.^[^ ^32^ ^]^

### 2.3 Visible Dynamics and Electron−Phonon Coupling

**Figure 3:**
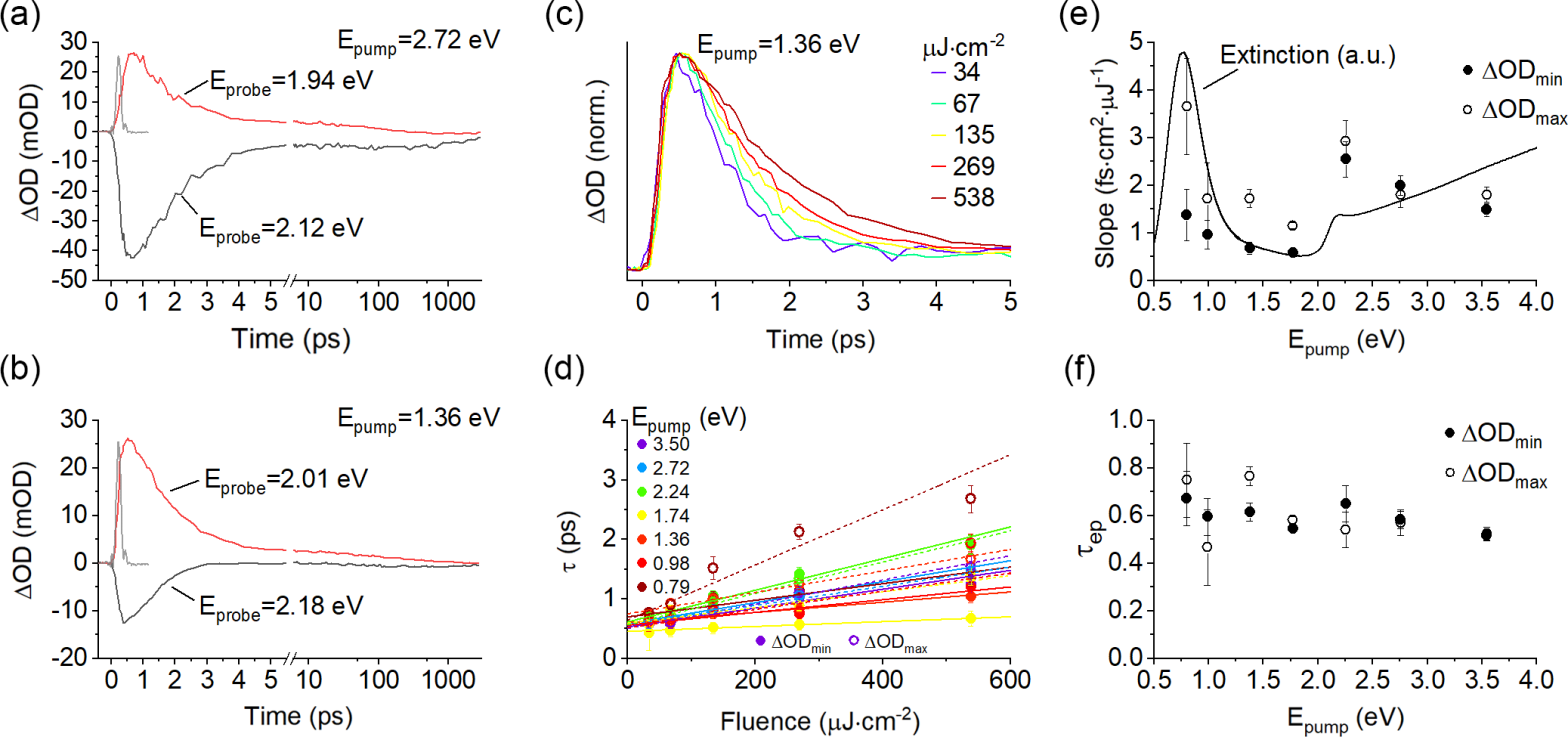
a,b) Temporal line‐cuts of the transient dynamics of copper NRs (NR 3) taken at minimum (black) and maximum (red) ΔOD values for a) 2.72 eV pump photon energy and b) 1.36 eV pump photon energy. The gray curve reflects the instrument response function as measured by Raman scattering by solvent. c) Normalized fluence‐dependent dynamics of a copper NR sample with 1.36 eV photon pump energy, probed at the peak‐induced absorption feature. d) Exponential fit decay values for fluence‐dependent dynamics for the same copper NR sample as a function of pump photon energy. Closed circles correspond to reduced absorption features; open circles correspond to induced absorption features. Dashed and solid lines in corresponding colors indicate a linear fit of the data points. The e) slope and f) extrapolated zero‐fluence value of the linear fit are plotted as a function of pump photon energy. In (e), the extinction spectrum of the sample is shown on an arbitrary scale alongside the measurement results. Error bars in all cases represent the standard deviation of fitting error.

The transient dynamics of plasmonic nanoparticles which are shown in **Figure** **3** may be generically broken into timescales corresponding to dephasing of the LSPR, electron−electron scattering, electron−phonon scattering, and phonon−phonon scattering.^[^ ^11^ ^]^ Representative dynamics traces for a copper NR sample are shown for interband excitation and intraband excitation in Figure 3a,b. The probe energies, which are labeled on the plots, correspond to the energies of ΔOD_min_ (black trace) and ΔOD_max_ (red trace), and the dashed gray line indicates the instrument response function of the measurement in this configuration, using Raman scattering of pure toluene. The temporal resolution of these experiments is too low to observe plasmon dephasing (<<100 fs), but electron−electron scattering may be observed from the rise time of the signals. A detailed picture of the timescale of the signal rise in the copper NR samples is shown in Figure S4, Supporting Information. The rise time of the signals, defined as the Gaussian full width at half maximum, associated with electron−electron scattering, are ≈350 fs for the copper NRs for intraband pumping but increase up to 550 fs for increasingly energetic interband excitation. This range of values is similar to the reported electron−electron scattering times in other coinage metals: 500−1000 fs for gold^[^ ^47^, ^50^, ^51^ ^]^ and 350 fs for silver.^[^ ^52^ ^]^

Electron−phonon coupling is apparent in the picosecond timescale decay of the transient optical response of the copper NRs in Figure 3a,b. As the electron gas dissipates heat to the lattice, the intensity of the transient optical response diminishes. As shown in the normalized dynamics traces in Figure 3c, the timescale of electron−phonon coupling is sensitive to the fluence of incident light, with slower response observed at higher photoexcitation intensities. It is typical in the literature to describe the decay of the transient extinction signal according to a two‐temperature model of coupled differential equations reflecting the temperature of the lattice ($T_{L}$) and the electrons ($T_{e}$)

(1) $$C_{e}\frac{\partial T_{e}}{\partial t}=-G(T_{L}-T_{e})$$

(2) $$C_{L}\frac{\partial T_{e}}{\partial t}=G(T_{e}-T_{L})$$

where $C_{e}$ and $C_{L}$ are the heat capacities of electrons and the lattice, respectively.^[^ ^53^ ^]^ Typically, this may be simplified such that the transient optical response may be fit to an electron−phonon coupling lifetime $\tau_{\text{ep}}\approx C_{e}/G$, in which $\tau_{\text{ep}}$ is an exponential decay time.^[^ ^11^ ^]^ Exponential fits to the transient dynamics of the copper NR samples were performed for each pump energy at probe energies corresponding to probe energies with $\Delta {\text{OD}}_{\text{max}}$ and $\Delta {\text{OD}}_{\text{min}}$ change in optical densities. The resulting fitted exponential time constants for one copper NR sample are plotted as a function of pump fluence in Figure 3d. As the exponential decay times vary substantially with fluence, the fundamental electron−phonon time may be estimated from the linear extrapolation of the fluence‐dependent decay times to the zero fluence limit.^[^ ^11^ ^]^ This approach permits a fluence‐independent comparison between pump excitation energies and samples which removes the strong dependence of the dynamics on pump fluence. The slope and extracted $\tau_{\text{ep}}$ values are plotted in Figure 3e,f, respectively; corresponding data for other samples are plotted in Figure S5, Supporting Information. Each copper NR sample showed, except for the lowest pump photon energy, similar slopes and electron−phonon coupling times, without systematic variation based upon the probe energy.

The slope of the linear fit was previously used to characterize the ease with which excitation at a given pump photon energy may be used to generate hot carriers.^[^ ^33^ ^]^ In this respect, two regimes are observed under interband and intraband excitation. Across all the samples, interband excitation closer to the bandgap energy yields a larger slope, which is surprising (if the slope reflects hot carrier generation) because the 2.24 eV photon energy pump can only photoexcite electrons to just above the Fermi energy. For intraband excitation, the slope of the *τ* versus fluence line increases with decreasing pump photon energy. Differences in the slope of the response should also reflect the absorption of the sample: weaker absorption will necessarily result in a relatively poorer signal for the same incident fluence. This appears particularly important in the intraband regime: larger slopes were generally observed with higher extinction. Indeed, peaks are observed near the onset of interband absorptions and at the longitudinal LSPR feature, as well as the smallest slope in each sample at 1.76 eV, where extinction is the weakest.

The electron−phonon coupling times $\tau_{\text{ep}}$ shown in Figure 3f (and Figure S5, Supporting Information) varied across all samples, pump energies, and visible probe energies over a range of ≈500 fs to 900 fs. As shown in **Table** **1**, $\tau_{\text{ep}}$ estimated for intraband excitation is somewhat longer (616 fs), on average, than under interband excitation (565 fs), but there is no clustering of $\tau_{\text{ep}}$ with pump photon energy as found in gold.^[^ ^33^ ^]^ Although previous work on noble metals^[^ ^54^, ^55^, ^56^ ^]^ and copper nanoparticles^[^ ^26^, ^28^ ^]^ showed an inverse size dependence of the electron−phonon coupling times, no distinctive size dependence was found among the copper NRs for the electron−phonon coupling times in Figure 3f and S5, Supporting Information. We attribute this to the relatively large NR size (short axes close to 20 nm) compared with earlier literature samples. Previous work found that electron−phonon coupling behavior in copper asymptotically approached bulk values above 20 nm diameter in quasispherical particles,^[^ ^26^ ^]^ a circumstance which also applies here. In addition, the twin boundaries of the NRs may also reduce the significance of size in electron−phonon coupling, as polycrystalline gold particles have shown size‐independent electron−phonon coupling behavior but single‐crystalline gold particles show size‐dependent behavior.^[^ ^57^ ^]^

**Table 1:** Average electron−phonon coupling times and constants of all copper NR samples for visible probe energies

Pump energy [eV]	*τ*_ep_ [fs]	*G*_Average_ [×10^16^ W m^−3^ K^−1^]
3.50	531 ± 27	5.5 ± 0.3
2.72	550 ± 38	5.3 ± 0.3
2.24	614 ± 76	4.8 ± 0.5
1.76	589 ± 28	5.1 ± 0.2
1.36	672 ± 44	4.5 ± 0.3
0.98	578 ± 10	5.1 ± 0.8
0.79	626 ± 14	5.0 ± 1.0

Average values of *G,* or the electron−phonon coupling constant, are given based upon the average $\tau_{\text{ep}}$ for each of the samples and conditions shown in Table 1, using literature values for the heat capacity of electrons in copper ($C_{e}=\gamma T$, where *γ* = 98.3 J · m^−3^ · K^−1^).^[^ ^53^ ^]^ Values for individual samples are shown in Table S1, Supporting Information. The *G* estimates calculated from $\tau_{\text{ep}}$ obtained at visible probe wavelengths show the similarity of electron−phonon coupling under the measured excitation conditions, ranging from 4.5 × 10^16^ to 5.5 × 10^16^ J m^−3^ K^−1^. Like other nanomaterials, they are also similar to the electron−phonon parameter in bulk copper from other sources, with reported values spanning from 2.2 × 10^16^ J m^−3^ K^−1^ to 1.0 × 10^17^ J m^−3^ K^−1^.^[^ ^16^, ^21^, ^22^, ^23^, ^24^, ^28^, ^58^, ^59^ ^]^ Thus, despite large differences in the spectral response under intraband and interband excitation, the dynamics of the copper NRs show little distinction between the excitation regimes. Most likely, this is because the dominant mechanism of electron−phonon coupling (e.g., via acoustic phonons) in the continuous density of states of the metal remains the same under all excitation conditions.

### 2.4 Transient Dynamics and Spectra at the Longitudinal LSPR Feature

**Figure 4:**
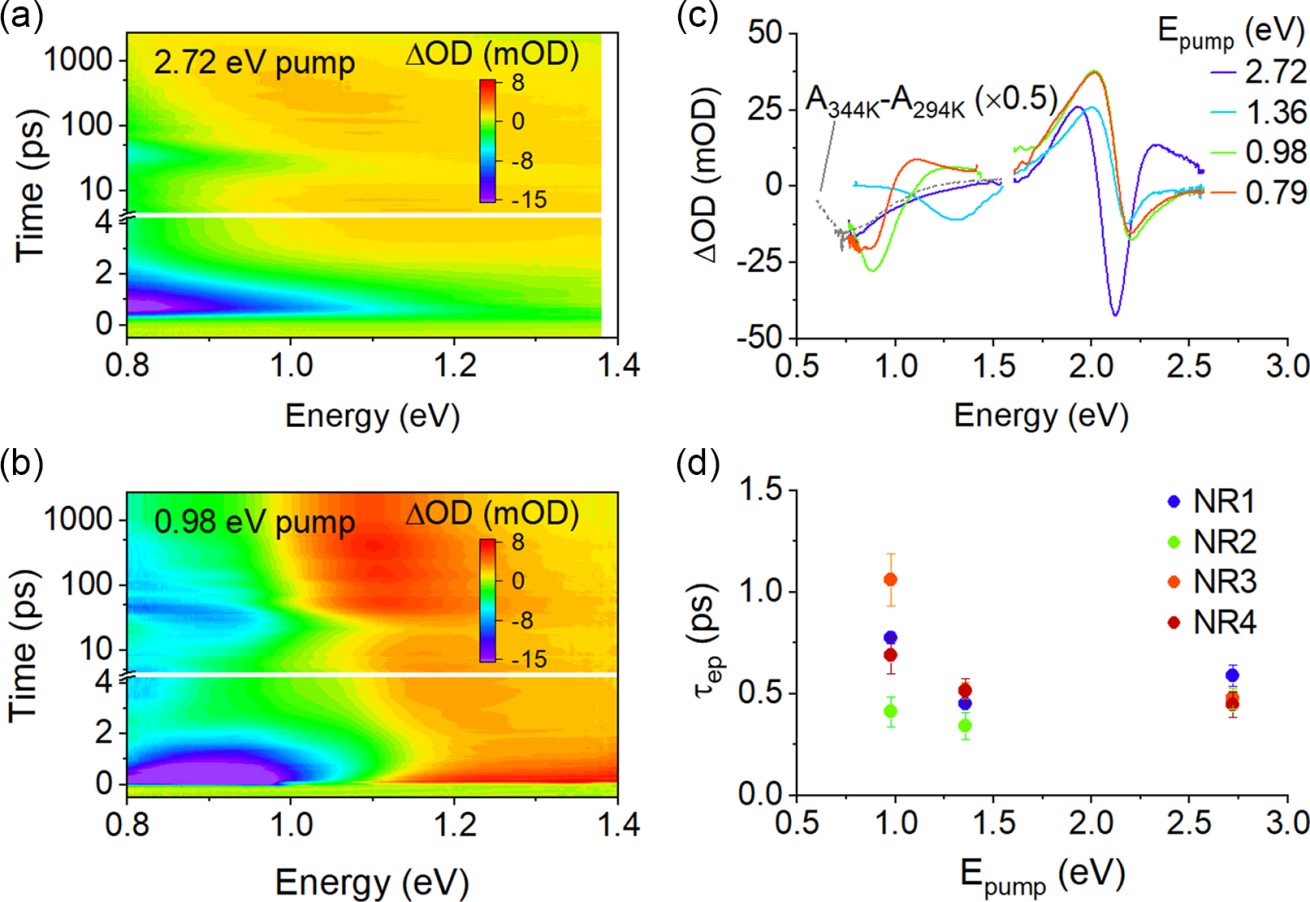
a,b) Map of the visible transient optical response of a copper NR sample (NR3) under excitation with a) 2.72 eV pump photon energy and b) 0.98 eV pump photon energy. c) Spectral response of the copper NR samples under photoexcitation at 538 μJ cm^−2^ for different photon pump energies. The gray dashed line shows the optical response of the sample from static thermal difference experiments. d) Extrapolated low‐fluence electron−phonon coupling times based upon the near‐infrared response of the copper NR samples. Error bars represent the standard deviation of fitting errors.

**Figure** **4** shows representative data on the near‐infrared response of copper NRs under interband (Figure 4a) and intraband (Figure 4b) excitations. With near‐infrared probe energies the optical response consists exclusively of the dynamics of the longitudinal LSPR feature. Under interband excitation at 2.72 eV, the extinction of the LSPR feature of the NRs is bleached. As shown in Figure 4c, this response is very similar to the change in extinction caused by static heating of the ensemble and likely originates from weakening (and potentially broadening) of the longitudinal LSPR. With intraband excitation on resonance with the LSPR feature, such as the 0.98 eV pump photon energy data shown in Figure 4b, the optical response is quite different: the extinction of the LSPR feature at energies greater than the pump energy increased and the extinction at lower energies decreases—that is, the LSPR feature undergoes a blueshift, which may also be accompanied by broadening due to increased optical losses. Each intraband pump energy results in an apparent blueshift of the LSPR feature centered at somewhat higher energies than the pump photon energy, as shown in Figure 4c. The spectral dependence of the response on the pump photon energy is more complex than the response at visible probe energies. It convolves fundamental material response with photoselection of the pump on the heterogeneous sample: different pump energies photoexcite different subpopulations of the ensemble. Although the underlying spectroscopic signature (a blueshift) is similar, the energy at which this feature is observed depends on the selected subpopulation of copper NRs which have an LSPR feature excited by the pump. This behavior does not exist under interband excitation. In that case, convergence with the result of ensemble static heating is consistent with interband photoexcitation of all copper NRs of the ensemble. Neither is photoselection apparent at visible probe energies (e.g., Figure 2 or Figure S2, Supporting Information) dominated by interband absorptions that are insensitive to sample size or morphology.

The origin of the LSPR blueshift derives from photoinduced changes of the carrier effective mass in the copper NRs. For a fixed medium and particle morphology, the energy of localized plasmon resonance is determined by underlying materials properties such as the carrier concentration (*N*) and carrier effective mass (*m* ^*^) via

(3) $$\omega_{\text{LSPR}}\propto \sqrt{\frac{Ne^{2}}{m^{*}\varepsilon_{0}\left(2\varepsilon_{m}+\varepsilon_{\infty }\right)}}$$

in which $\varepsilon_{\infty }$ is the high‐frequency dielectric constant of the material and $\varepsilon_{m}$ is the medium dielectric function. The carrier concentration (*N*) is not immediately impacted by intraband excitation and volumetric expansion of the lattice which decreases *N* occurs only after electron‐phonon coupling. Therefore, to explain the observed blueshift of the LSPR feature, the effective mass of the carriers in the copper NRs must be decrease at higher electronic temperatures under photoexcitation. Indeed, although fitting of the optical effective mass in copper as a function of temperature is limited, one report indicates decrease in *m* ^*^ of copper from 1.49 at 298 K to 1.43 at 423 K.^[^ ^29^ ^]^ Those (static) measurements provide a strong indication that the effective mass of copper under higher electronic temperatures is also expected to decrease, consistent with the observed LSPR blueshift.

The picosecond timescale dynamics of the copper NR samples in the near–infrared region are similar to visible probe energies. As at visible probe energies, fluence‐dependent dynamics for various pump energies are fitted with exponential decays to provide other estimates of electron−phonon coupling times shown in Figure 4d, with corresponding *G* estimates in **Table** **2** (unaveraged data are shown in Table S2, Supporting Information). Here, for 2.72 eV pump, the probe energy was chosen as the minimum ΔOD signal; for intraband excitation, the probe energy for fitting ΔOD dynamics was chosen 50 meV below the pump energy. Consistent with the common origin in the ΔOD signal with visible response, the resulting $\tau_{\text{ep}}$ and *G* are similar, albeit more variable.

**Table 2:** Average electron−phonon coupling times and constants of copper NR samples at near‐infrared probe energies

Pump energy [eV]	*τ*_ep_ [fs]	*G*_Average_ [×10^16^ W m^−3^ K^−1^]
2.72	496 ± 52	5.9 ± 0.6
1.36	455 ± 45	6.4 ± 0.6
0.99	733 ± 80	4.4 ± 0.5

### 2.5 Coherent Acoustic Phonons

**Figure 5:**
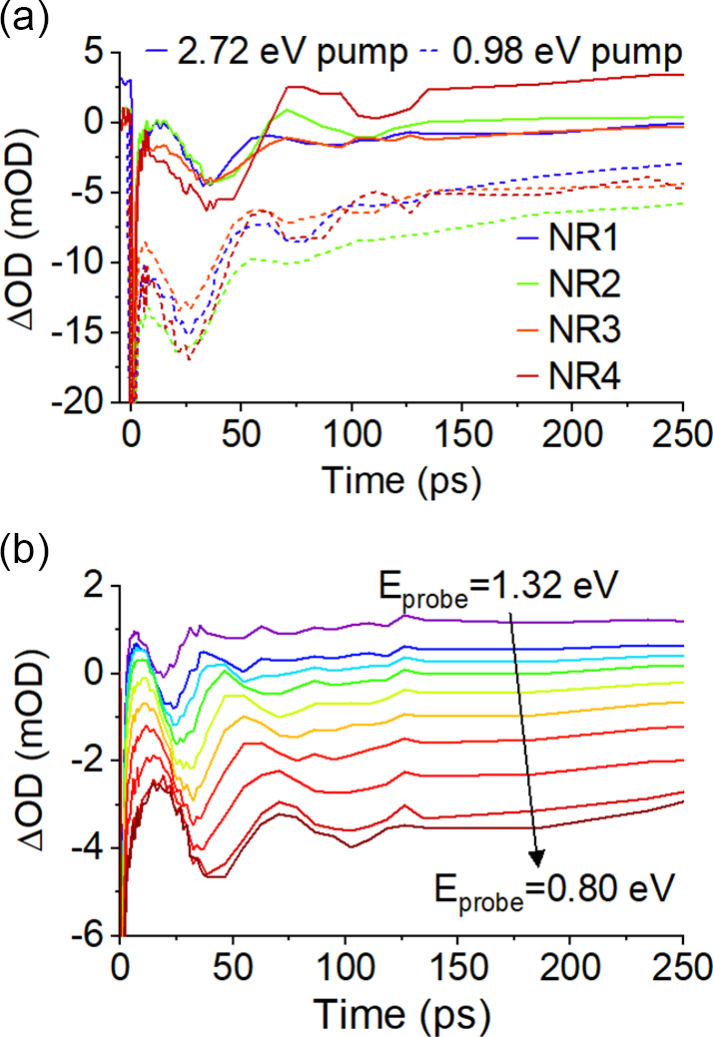
a) Offset temporal line‐cuts at near‐infrared probe energies for four copper NR samples with pump energies of 2.72 eV (solid lines) and 0.98 eV (dashed lines). b) Temporal dynamics as a function of probe photon energy for a copper NR (NR 3) sample photoexcited with 538 μJ cm^−2^ pump with photon energy 2.72 eV.

After the equilibration of the lattice and electron temperature through electron−phonon coupling, a slower process occurs in which hot particles dissipate heat to the surrounding environment—here toluene solvent—through phonon−phonon coupling. Typically, this process occurs over tens to hundreds of picoseconds. The data in Figure 2a,b and 3a,b demonstrate this type of slow dissipation at longer pump‐probe delay times. However, under certain circumstances, namely, when the lattice heating of a nanoparticle is faster than the period of vibrational modes associated with thermal expansion, individual acoustic phonon modes may be observed spectroscopically. Coherent acoustic phonons consisting of the collective nuclear motion of entire nanoparticles have been observed in ensembles of noble metal nanoparticles,^[^ ^60^, ^61^, ^62^, ^63^ ^]^ quantum dots,^[^ ^64^, ^65^ ^]^ or measurements of individual nanostructures.^[^ ^66^, ^67^ ^]^ Experimentally, coherent acoustic phonons are detected from sinusoidal temporal signatures, typically superimposed on a global signal decay. Such acoustic phonons are strongly sensitive to local environments and they have been proposed for highly sensitive detection of mass and solvent properties.^[^ ^68^, ^69^, ^70^ ^]^ They are also highly dependent on the elastic properties and physical dimensions of the sample and the ensemble dispersion, for which the different oscillatory frequencies of subpopulations, ensemble polydispersity of size, lead to dephasing.^[^ ^55^ ^]^ Spherical impurities, which can contribute to the visible spectral response, are also be expected to lead to destructive interference. For the copper NRs in this study, such coherent oscillatory signals were not observed in experiments probing at visible probe energies. However, they are apparent in Figure 4a,b at energies corresponding to the longitudinal LSPR mode. This finding was common to all the measured samples, as shown in **Figure** **5**a. Each copper NR sample showed at least one period of the sinusoidal oscillation of ΔOD under interband and intraband excitation, but these were damped or no longer apparent after no more than two periods. Of note, the observed period of the oscillation depends strongly on the probe photon energy (Figure 5b), with higher probe photon energies showing shorter periods, changing from 31 ps at 1.32 eV to 56 ps at 0.80 eV. Variation in acoustic phonon periods stems from distribution of NR aspect ratio: the higher frequency at higher probe energies is from shorter copper NRs with higher‐energy extensive phonon modes.^[^ ^11^, ^55^ ^]^

## 3 Conclusion

In conclusion, ultrafast pump‐probe measurements on copper NRs have shown a strong pump energy dependence of the transient optical response, depending on whether the excitation excites interband or intraband transitions. Despite the large spectral dependence on pump photon energy, the temporal dynamics are relatively similar for all pump energies, decreasing modestly with increases in pump photon energy. Electron‐phonon coupling in copper NRs is similar to bulk copper. The transient optical response of the samples at the LSPR is complicated by photoselection effects and presence of acoustic phonons apparent in the optical signal, but resonant excitation leads to a transient LSPR blueshift. In timescale, these results are similar to earlier work on gold and silver in both bulk and nanoscopic form. But the stark differences in the spectral response shown here contrasts starkly with other coinage metals, which offers important guidance for use of different excitation schemes in photochemistry or optical devices and shows that copper and nanostructures made from copper have a distinct place among the coinage metals.

## 4 Experimental Section

4.1

4.1.1

##### Chemicals

Gold(III) chloride trihydrate (HAuCl_4_·3H_2_O, 99.9%), copper(II) chloride dihydrate (CuCl_2_·2H_2_O, 99.999%), oleylamine (OLAM‐SA, 70%), borane *tert*‐butylamine complex (TBAB, 97%), acetone (99.5%), and 2‐propanol (IPA, 99.5%) were purchased from Sigma Aldrich. Oleylamine (OLAM‐TCI, 50%) was purchased from TCI America. Anhydrous toluene and tetrahydrofuran (THF) were obtained from a custom‐built solvent purification system. OLAM‐SA for copper NR synthesis was used as received without further purification. OLAM‐TCI for Au NP seed synthesis was pre‐dried at 100 °C under vacuum for 4 h and stored inside a N_2_‐filled glovebox before use.

##### Synthesis of Au NC Seeds

In a typical synthesis of 9 nm Au NC seeds, 10 mL of OLAM‐TCI was loaded in a 50 mL three‐neck flask. After degassing under vacuum for 20 min at room temperature, 10 mL of anhydrous toluene was injected into the flask followed by flushing with N_2_ for 10 min. Afterward, 0.25 mmol (98 mg) of HAuCl_4_·3H_2_O was added into the mixture followed by purging with N_2_ for another 20 min. The solution temperature was maintained at 15 °C by ice bath. Subsequently, a solution mixture of 0.25 mmol (22 mg) of TBAB, 1 mL of OLAM‐TCI, and 1 mL of anhydrous toluene was swiftly injected. The resulting mixture was kept under stirring for another 1 h at room temperature. Au NCs were isolated via precipitation with 60 mL of acetone followed by centrifugation at 6000 rpm for 5 min. The NC precipitates were redispersed in anhydrous toluene to attain an optical density (OD) of 40 at the peak LSPR wavelength.

##### Synthesis of Copper NRs

Copper NRs were synthesized following our previously reported method.^[^ ^15^ ^]^0.5 mmol (85 mg) of CuCl_2_·2H_2_O was mixed with 10 mL of OLAM‐SA in a 50 mL three‐neck flask. The mixed solution was evacuated under vacuum at 25 °C followed by N_2_ gas flushing for three times. Afterward, the solution was heated at 80 °C for 1h to fully dissolve CuCl_2_, resulting in blue color solution. Next, the solution was heated to 180 °C, to produce a yellowish solution when a certain amount of Au NC seed solution (OD = 40) was injected. The reaction solution was kept at 180 °C for 1 h. For NR1 (5.1 ± 1.4 nm), NR2 (7.0 ± 2.8 nm), NR3 (8.4 ± 1.9 nm), and NR4 (8.9 ± 1.3 nm), 0.35, 0.3, 0.25, and 0.2 mL of Au seed solution was injected, respectively. After cooling down to room temperature, the NR products were collected by precipitation with 30 mL IPA followed by centrifugation at 3000 rpm for 3 min. The NRs were finally dispersed in anhydrous toluene and stored inside a N_2_‐filled glovebox.

##### Polymer Functionalization of Copper NRs

Copper NRs were grafted with polystyrene‐pentaethylenehexamine (PS‐PEHA) to improve colloidal stability. PS‐PEHA was synthesized in house according to our previously reported method.^[^ ^71^ ^]^ To functionalized copper NRs with PS‐PEHA, 20 mg of 5.6 k PS‐PEHA was dissolved in 1.6 mL of anhydrous THF, to which 0.5 mL of copper NR solution (5 mg mL^−1^ in toluene) was added. The solution was sonicated for 10 s and was left undisturbed for 12 h inside a glovebox. Afterward, copper NRs were retrieved via precipitation with anhydrous heptane followed by centrifugation at 3000 rpm for 3 min. The precipitates were redispersed in toluene or tetrachloroethylene (TCE).

##### Characterization

Low‐magnification TEM images were acquired on a JEOL JEM 1400 plus microscope equipped with a LaB_6_ filament operating at 120 kV. TEM samples were prepared by drop‐casting ≈10 μL of NC solution onto 300‐mesh carbon‐coated copper grids (Ted Pella). UV‐Vis‐NIR extinction spectra were acquired on a Varian Cary‐5000 UV‐Vis‐NIR spectrophotometer (Agilent).

##### Spectroscopy

All measurements, both steady‐state and transient, were performed using air‐sealed 2 mm cuvettes which were loaded in a nitrogen glovebox with the sample dispersed in anhydrous toluene. Samples were loaded with magnetic stir bars and stirred during measurements. Steady state spectroscopy, including temperature‐dependent extinction measurements, were performed using two spectrometers. Ultraviolet to near infrared measurements to ≈1.2 eV were performed using a Cary‐60 spectrophotometer. Static measurements from 1.5 to 0.5 eV were performed using a Nicolet 6700 FT‐IR. For temperature‐dependent extinction measurements, samples were loaded into a cuvette heating block with a spacer for the thinner cuvettes and permitted to equilibrate for 15 min after reaching set point temperatures. Control measurements were also performed using blank toluene cuvettes to observe any changes in the transmittance of the solvent with temperature.

Transient spectroscopic measurements of the samples were performed by splitting the fundamental output of an amplified, 60 fs Ti: sapphire laser operating at 5 kHz. A pump beam was generated from one branch, using an optical parametric amplifier to generate the different photoexcitation energies used in this work. The energies given for the pump beams in this work were determined from the peak of the scattering spectrum (see Figure S1, Supporting Information). The pump beam was directed through a mechanical chopper to yield a 2.5 kHz source which was focused on to the samples using appropriate lenses. The second branch of the fundamental was routed through a mechanical delay stage and focused into a sapphire crystal to generate a supercontinuum white light to function as the broadband probe. For visible probe energies, a 1 mm sapphire crystal was used; for near infrared probe energies, a 5 mm crystal was used. The pump and probe were focused on to the sample and spatially overlapped for measurements.

##### Data Fitting

Transient absorption data were fitted using SurfaceXplorer software using monoexponential decays convolved with a Gaussian rise feature.

(4) $$\Delta A(t)=e^{{\left(\frac{2\ln2\left(t-t_{0}\right)}{t_{IRF}}\right)}^{2}}\cdot \Delta A_{0}e^{\frac{t-t_{0}}{\tau }}$$

Only data from the first 10 ps of pump‐probe delay was fit to estimate $\tau_{ep}$.

## Conflict of Interest

The authors declare no conflict of interest.

## Supporting information

Supplementary Material

## Data Availability

Research data are not shared.
