# Supplementary material for: Ultrafast Dynamics of Colloidal Copper Nanorods: Intraband versus Interband Excitation
Source: Small Sci. 2021 Dec 15;2(3):2100103. doi: 10.1002/smsc.202100103 (PMC11935956; doi:10.1002/smsc.202100103)
Supplement: Supplementary file 1 — Supplementary Material [file SMSC-2-2100103-s001.pdf]

Supporting Information

Ultrafast Dynamics of Colloidal Copper Nanorods: Intraband versus Interband Excitation

Dr. Benjamin T. Diroll, * *Soojin Jeong, and Prof. Xingchen Ye* *

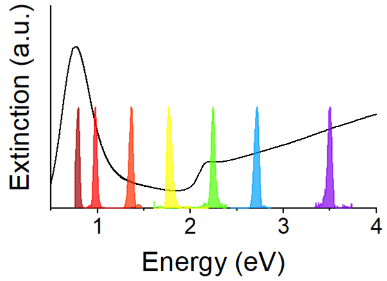

Figure S1. Extinction spectrum of a copper NR sample (NR3) overlaid with scattering spectra of different pump excitations used in the work.

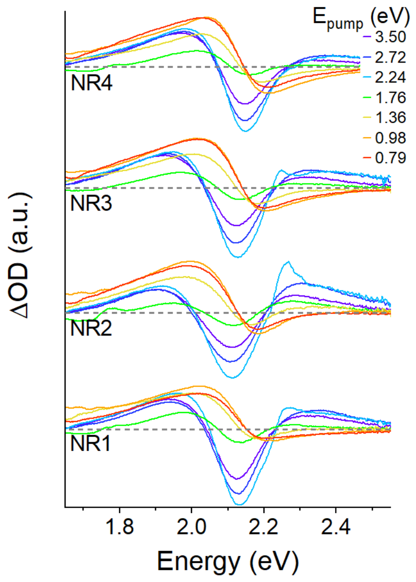

Figure S2. Transient extinction spectra of four copper NR samples collected at a pump-probe delay of 500 fs for different pump excitation energies as labeled in the key. The fluence of each pump excitation was maintained at 538 μJ·cm^{-2}. Dashed lines indicate a value of ΔOD=0 for each set of spectra.

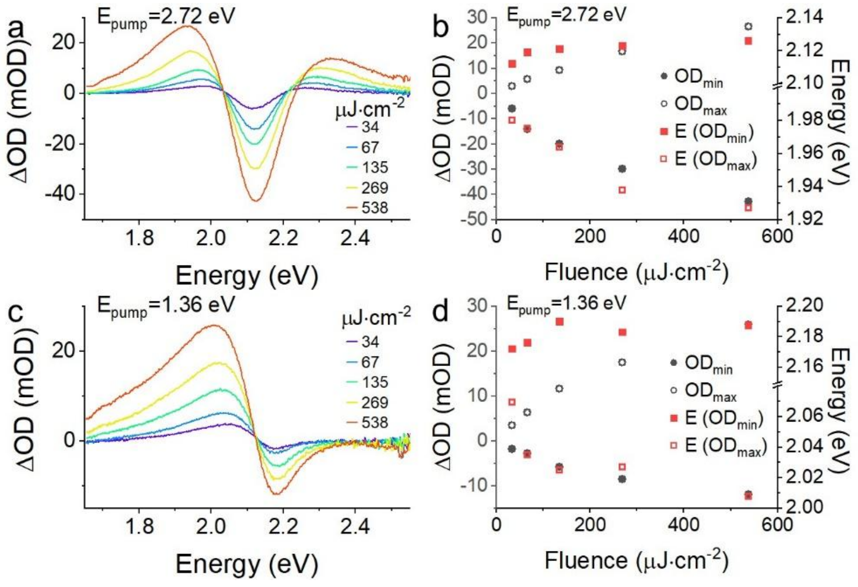

Figure S3. (a) Pump-fluence-dependent visible transient extinction spectra of a copper NR sample (NR3) with 2.72 eV pump excitation. (b) ΔOD_{min} and ΔOD_{max} values (left axis), as the corresponding energy of the peak extinction change values of the samples with 2.72 eV pump excitation. (c) Fluence-dependent visible transient extinction of the same NR sample with 1.36 eV pump excitation and corresponding (d) maximum and minimum ΔOD and energies.

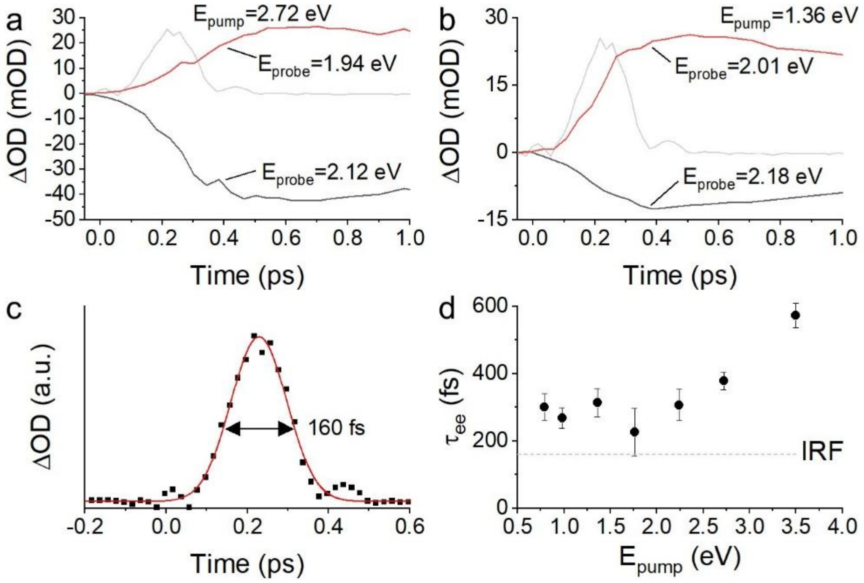

Figure S4. (a, b) Time-traces at specified probe energies of transient extinction measurements of a copper NR ensemble under (a) 2.72 eV photon energy pump and (b) 1.36 eV photon pump energy. The gray line represents the instrument response function, which is also shown in (c). The instrument response was derived from the Raman scattering signal of toluene solvent. (d) Fitted gaussian rise times of the copper NR sample as a function of pump photon energy. Error bars indicate the standard deviation of fitting error.

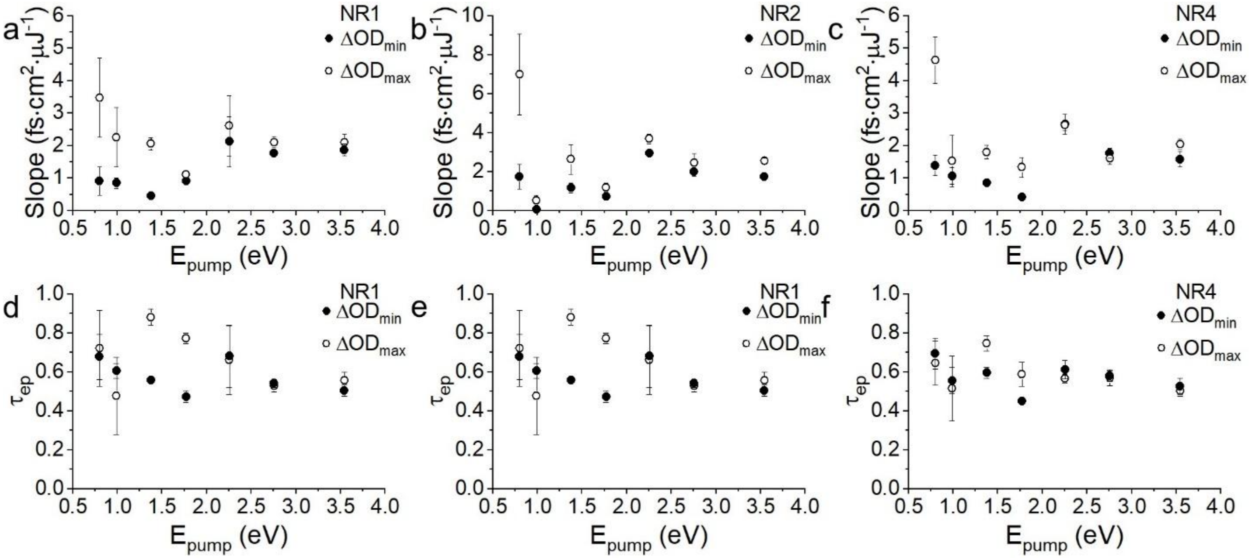

Figure S5. (a-c) Extracted slopes of τ versus fluence for different pump photon energies for samples (a) copper NR1, (b) NR2, and (c) NR4. (d-f) Extracted τ_{ep} versus fluence for different pump photon energies for samples (d) copper NR1, (e) NR2, and (f) NR4. Error bars in all cases represent the standard deviation of fitting error.

Table S1. Extracted Electron Phonon Coupling Parameters for the given samples and pump energy conditions, using visible probe wavelengths.

Pump Energy (eV)	G_{NR1}[a]	G_{NR2}	G_{NR3}	G_{NR4}
3.50	5.5±0.3	5.2±0.2	5.6±0.2	5.6±0.3
2.72	5.4±0.3	5.5±0.3	5.5±0.3	5.5±0.3
2.24	4.3±0.8	4.8±0.2	4.9±0.6	4.9±0.3
1.76	4.9±0.3	4.5±0.2	5.2±0.1	5.7±0.3
1.36	4.2±0.1	5.2±0.5	4.2±0.2	4.4±0.2
0.98	5.4±1.0	4.1±0.2	5.5±1.1	5.4±1.0
0.79	4.2±0.7	7.3±2.3	4.1±0.7	4.3±0.5

[a]All electron-phonon coupling constants given in (10^{16} W·m^{-3}·K^{-1})

Table S2. Extracted Electron Phonon Coupling Parameters for the given samples and pump energy conditions, using near-infrared probe wavelengths.

Pump Energy (eV)	G_{NR1}[a]	G_{NR2}	G_{NR3}	G_{NR4}
2.72	4.9±0.4	6.2±0.5	6.1±0.6	6.5±0.8
1.36	6.4±0.2	8.5±1.4	5.6±0.4	5.7±0.6
0.99	3.7±0.1	7.1±1.1	2.7±0.3	4.2±0.5

[a]All electron-phonon coupling constants given in (10^{16} W·m^{-3}·K^{-1})
